# Compression of the Left Atrium and Left Ventricle by a Thoracoabdominal Aortic Aneurysm: A Case Report

**DOI:** 10.3400/avd.cr.20-00092

**Published:** 2020-12-25

**Authors:** Koichi Nagaya, Masaaki Naganuma, Yasushi Kudoh, Nobuaki Suzuki, Shinya Masuda

**Affiliations:** 1 Department of Cardiovascular Surgery, Aomori Prefectural Central Hospital, Aomori, Aomori, Japan

**Keywords:** thoracoabdominal aortic aneurysm, compression, cardiac failure

## Abstract

We describe the case of a 66-year-old man with a thoracoabdominal aortic aneurysm, who presented with cardiac failure; he had complained of shortness of breath. A contrast-enhanced computed tomography scan and transthoracic echocardiography showed compression of the left atrium and ventricle by a giant thoracoabdominal aortic aneurysm. The cardiac failure resolved after early prosthetic graft replacement surgery.

## Introduction

A thoracoabdominal aortic aneurysm (TAAA) presenting with symptoms of cardiac failure due to compression of the surrounding structures is a rare phenomenon. Here we present a case of a giant TAAA in a patient who presented with cardiac failure due to compression of the left atrium and ventricle.

## Case Report

A 66-year-old man, with a history of graft replacement for an abdominal aortic aneurysm, presented at another hospital with shortness of breath that had gradually worsened over the last two months. The degree of dyspnea on admission corresponded to New York Heart Association functional class II. He was transferred to our hospital after a computed tomography scan revealed a giant TAAA. On physical examination, his blood pressure was 125/87 mmHg, heart rate was 98 bpm, and oxygen saturation was 94%. Laboratory findings showed renal dysfunction with elevated serum creatinine to 2.03 mg/dL (normal range 0.65–1.07 mg/dL) and brain natriuretic peptide to 75.4 pg/dL (normal range <18.4 pg/dL).

**Figure 1:**
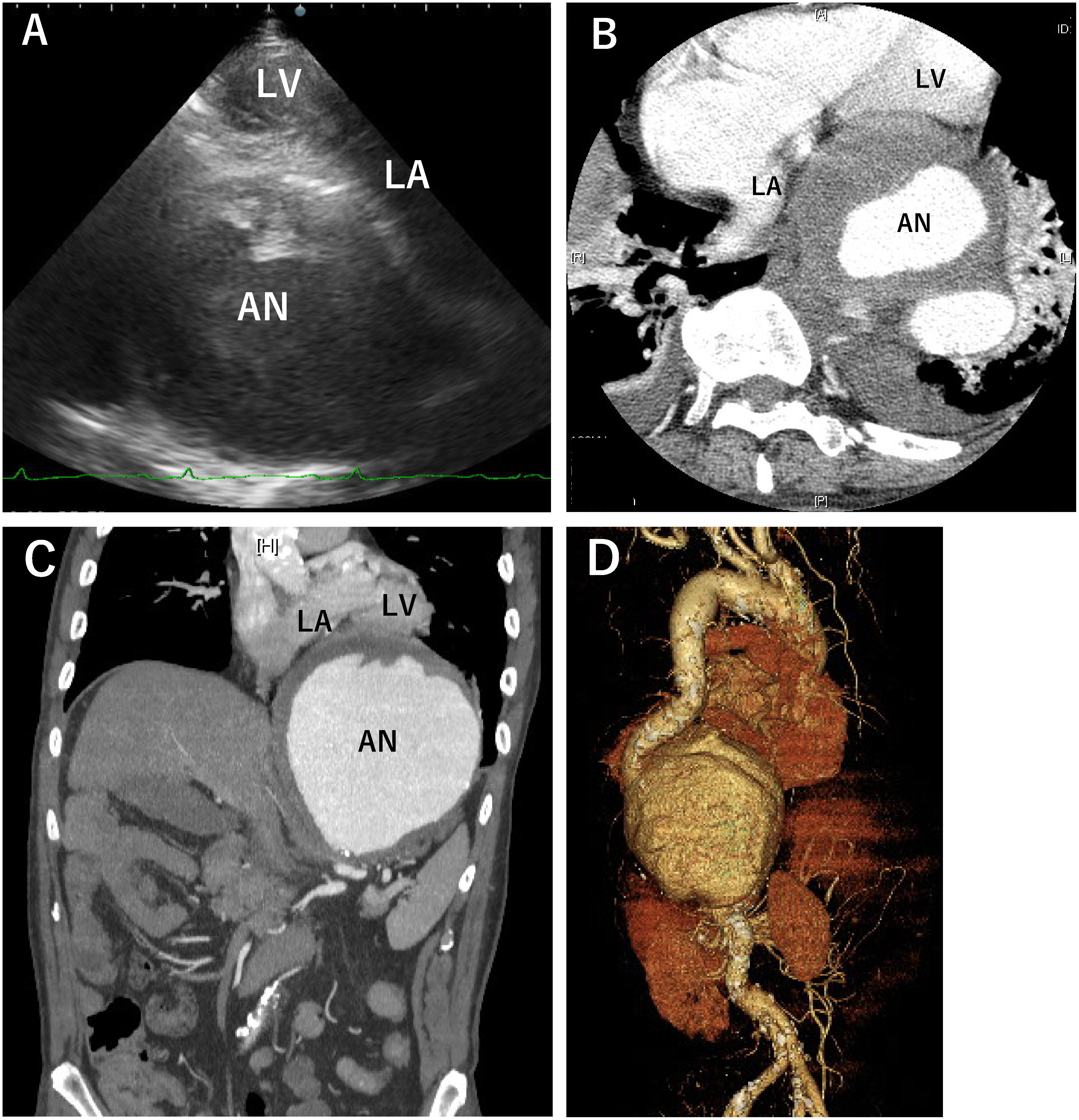
Fig. 1 Preoperative transthoracic echocardiography (**A**) and contrast-enhanced computed tomography in the axial (**B**) and coronal (**C**) planes showing compression of the LA and LV by a thoracoabdominal aortic aneurysm. Three-dimensional computed tomography image (**D**) showing a giant aneurysm in the thoracoabdominal aorta measuring 140 mm in diameter.

LA: left atrium; LV: left ventricle; AN: aortic aneurysm

The electrocardiogram showed a normal sinus rhythm without ST changes. A chest X-ray revealed cardiomegaly with a cardiothoracic ratio of 62%: the TAAA was not visible as it was masked by the cardiac shadow. Transthoracic echocardiography (TTE) showed that the left atrium (LA) and left ventricle (LV) were compressed by the TAAA (**Fig. 1A**). Valve regurgitation was not detected. The left ventricular systolic function was normal with an ejection fraction of 60%, and the left ventricle end-diastolic dimension (LVDd) was 34 mm as observed on the M-mode echocardiogram. On pulsed-wave Doppler imaging, E/A (E: early diastolic transmitral flow velocity; A: late diastolic transmitral flow velocity) was 0.66, and on pulsed-wave tissue Doppler imaging, E/e′ (e′: early diastolic mitral annular velocity) was 17.10.

**Figure 2:**
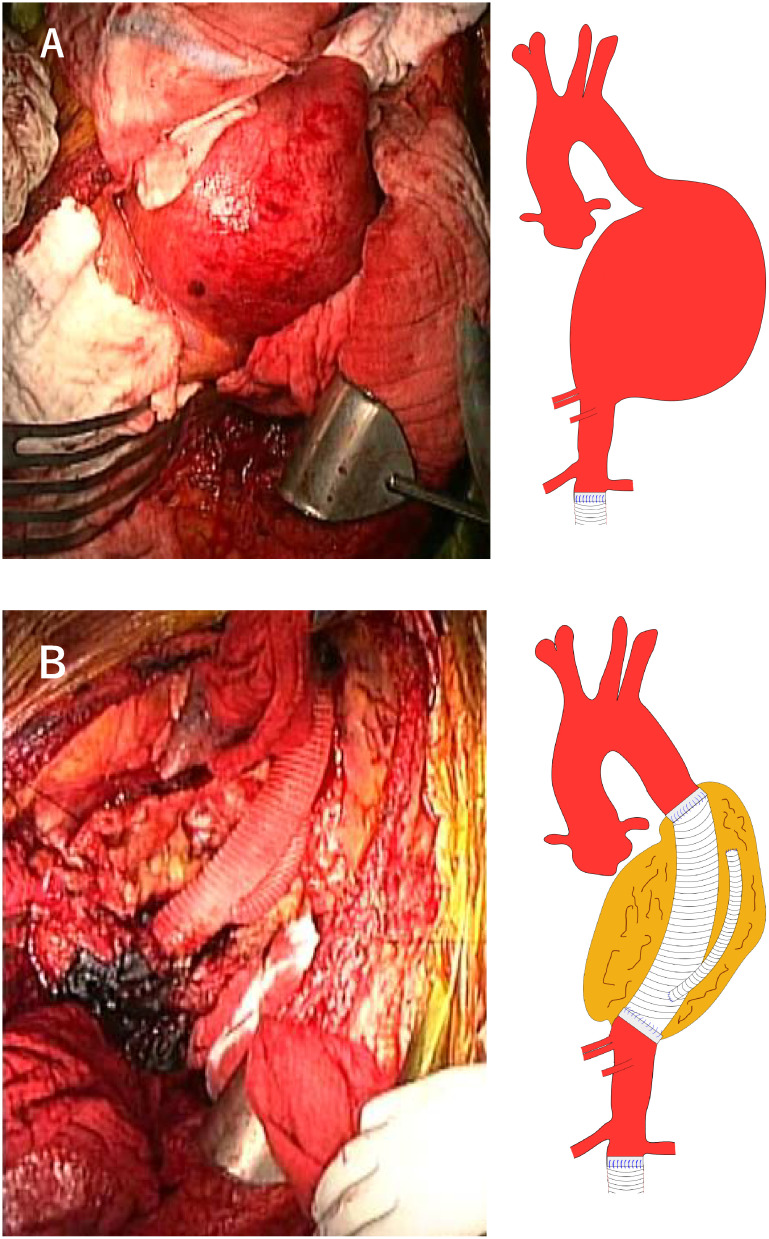
Fig. 2 Intraoperative findings and schematic demonstration. A giant aneurysm involving the thoracoabdominal aorta (**A**) was identified. Graft replacement using a 22 mm Dacron graft was performed (**B**).

A contrast-enhanced computed tomography (CECT) scan revealed compression of the LA and LV by a giant aneurysm in the thoracoabdominal aorta measuring 140 mm in diameter. The aneurysm affected the aorta from the level of the ninth thoracic vertebra to the celiac artery (type V according to the Crawford–Safi classifications) (**Figs. 1B**–**1D**). The artery of Adamkiewicz was not visualized. Four days after admission to our hospital, the patient underwent urgent surgery due to the size of the aneurysm (giant) and aggravation of symptoms. An attempt to insert a cerebrospinal fluid drainage catheter on the day before surgery to protect the spinal cord was unsuccessful because of the fusion of the vertebrae. Under general anesthesia with differential lung ventilation, a spiral incision was performed. He was placed on partial cardiopulmonary bypass through the cannulation of the common femoral artery and vein. Graft replacement, using a Dacron graft (22 mm; J-graft; Japan Lifeline Inc., Tokyo, Japan), was performed with reconstruction of the ninth intercostal artery and beveling anastomosis of the distal site (**Figs. 2A** and **2B**). The surgical duration was 460 min, the duration of cardiopulmonary bypass was 118 min, and the bleeding volume was 2911 mL.

**Figure 3:**
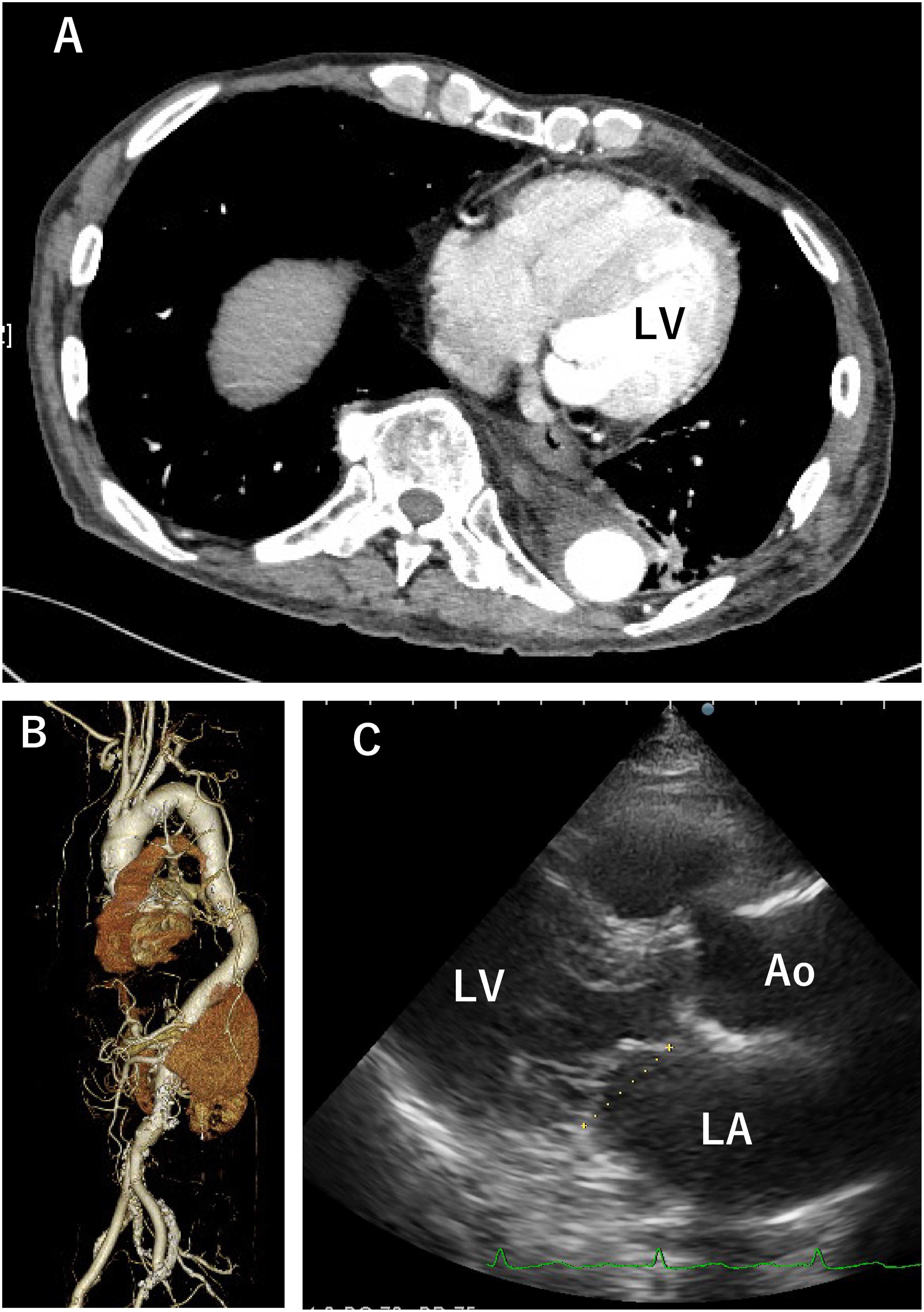
Fig. 3 Postoperative contrast-enhanced computed tomography (**A**, **B**) and transthoracic echocardiography (**C**) showing a well-repaired thoracoabdominal aorta and the LA and LV released from the compression of aortic aneurysm.

LA: left atrium; LV: left ventricle; Ao: aorta

He was extubated on postoperative day (POD) 1 with no neurological deficits but had to be reintubated on POD 5 due to acute respiratory distress syndrome and pneumonia. He also required hemodialysis for 20 days after surgery due to renal failure. Thereafter, his respiratory failure and renal dysfunction gradually improved, and he was transferred to another hospital for rehabilitation on POD 58; the cardiac failure had resolved by then. Postoperative CECT and TTE showed a well-repaired thoracoabdominal aorta (**Figs. 3A** and **3B**), and the LA and LV were not compressed (**Fig. 3C**).^1)^ At a two-year post-surgery follow-up visit, the patient was doing well without any signs of cardiac failure or aggravated renal failure that would require hemodialysis.

## Discussion

Most patients with an aortic aneurysm are asymptomatic, and the aneurysm is usually discovered accidentally on chest radiography, echocardiography, computed tomography, or magnetic resonance imaging.^1,2)^ Aneurysms that present with symptoms are typically very large and are prone to rupture, which is associated with a high rate of mortality. In symptomatic cases, patients might complain of chest or back pain, or symptoms related to compression of the surrounding structures (trachea, bronchus, esophagus, superior vena cava, left and right atrium or pulmonary veins).^3–5)^

In our patient, the recent worsening of symptoms suggested rapid dilatation of the aneurysm, necessitating urgent surgical intervention. Therefore, we were unable to perform further evaluation, including a pressure study before surgery.

Although it is possible that the right heart load generated by the compression of the right heart system contributed to cardiac failure, we believe that the primary cause of cardiac failure was the compression of the LA and LV by TAAA (shown on CECT and TTE), which pushed the whole heart upward and forward. In such a situation, cardiac failure develops due to increased pulmonary capillary wedge pressure and impaired LV diastolic filling due to compression of the LA.^6,7)^ In our case, it was doubtful whether parameters indicative of diastolic dysfunction were visible; however, we considered that a high E/e′ ratio suggested an increased left atrial pressure and diastolic dysfunction. We believe that the diastolic dysfunction may have resulted from LV compression as visualized on TTE.

Compression of the LA due to a TAA or TAAA has been previously described.^6–8)^ However, compression of both the LA and LV by TAAA is rare. To our knowledge, this is the first case of cardiac failure caused by the compression of both the LA and LV.

## Conclusion

In this report, we successfully treated a very rare case of cardiac failure caused by compression of the LA and LV due to a TAAA, which was diagnosed by TTE in combination with CECT. Surgical decompression of the aneurysmal sac and graft replacement can effectively improve cardiac failure in similar cases.

## Additional Note

We have obtained informed consent from the patient.
